# Seamless 5G Multi-Hop Connectivity Architecture and Trials for Maritime Applications

**DOI:** 10.3390/s23094203

**Published:** 2023-04-22

**Authors:** Artürs Lindenbergs, Maciej Muehleisen, Miquel Payaró, Kati Kõrbe Kaare, Helmut W. Zaglauer, Johan Scholliers, Arvi Sadam, Kristjan Kuhi, Lasse Nykanen

**Affiliations:** 1 Latvijas Mobilais Telefons SIA, 6 Ropazu Street, LV-1039 Riga, Latvia; arturs.lindenbergs@lmt.lv; 2 Ericsson GmbH, Ericsson-Allee 1, 52134 Herzogenrath, Germany; maciej.muehleisen@ericsson.com; 3 Centre Tecnològic de Telecomunicacions de Catalunya (CTTC/CERCA), Parc Mediterrani de la Tecnologia, Castelldefels, 08860 Barcelona, Spain; 4 Department of Mechanical and Industrial Engineering, Tallinn University of Technology (TalTech), Ehitajate tee 5, 19086 Tallinn, Estonia; kati.korbe@taltech.ee (K.K.K.); kristjan.kuhi@taltech.ee (K.K.); 5 Airbus Defence and Space GmbH, Claude-Dornier-Strasse, 88090 Immenstaad, Germany; helmut.zaglauer@airbus.com; 6 VTT Technical Research Centre of Finland, P.O. Box 1300, 33101 Tampere, Finland; johan.scholliers@vtt.fi; 7 Ericsson Eesti, Valukoja 8, 11415 Tallinn, Estonia; arvi.sadam@ericsson.com; 8 Vediafi Ltd., Valimotie 13A, 00380 Helsinki, Finland; lasse.nykanen@vedia.fi

**Keywords:** 5G, seamless connectivity, maritime connectivity, multi-hop, private networks, hybrid terrestrial–satellite, non-terrestrial networks

## Abstract

This paper provides a study of the different alternatives that are being considered in the 5G-ROUTES project to establish seamless 5G connectivity in a maritime environment both from an architectural point of view and also from the definition of field trials to evaluate the performance and dependability of the proposed solution. As expected, the main challenge in providing 5G connectivity on the sea is to provide coverage over large areas of open water. Thus, as a starting point, this paper presents a measurement campaign that was conducted to assess the current coverage in the Baltic Sea, which concluded that the current terrestrial networks cannot guarantee sufficient coverage. Next, the solution architecture and trials proposed by 5G-ROUTES are described, which are based on the integration of satellite and leading-edge multi-hop connectivity in 5G networks. Utilizing satellite backhaul can potentially overcome the connectivity challenge from the terrestrial domain to the maritime domain, while multi-hop connectivity ensures that coverage is extended among the different ships that are navigating the sea. Furthermore, this paper describes how the project will evaluate, in field trials tailored to this maritime environment, common connectivity key performance indicators (KPIs) such as latency, throughput, availability and reliability. This paper concludes by providing a vision for applying the obtained results and insights to maritime transportation and other remote areas where the deployment of a suitable 5G infrastructure may be challenging or costly. The findings will be used to guide the design of future 5G networks for marine applications and to identify the most effective methods for providing secure and dependable communication in a maritime setting.

## 1. Introduction

The exploitation of oceans can enable huge economic growth, job creation, and innovation. For example, Ref. [1] showed that, by 2030, the contribution of the marine economy to global value added will be in the range of US$3 trillion, creating about 40 million jobs in the process. The maritime sector is frontrunning regarding digitalization and onboard information and communication technology (ICT) usage onboard. Major technical inspections (class renewal), typically carried out every five years, are often used for the substantial modernization of ICT systems. However, radio communication, besides analogue voice, satellite and WiFi, has only received limited attention until now. Fifth generation (5G) and sixth generation (6G) mobile communications bear the promise of rolling out seamless connectivity over the sea with a direct impact on the quality of life and quantity of service for labor forces involved in shipping and logistics. A major legislation push towards a new era of digitalization is forcing the maritime cluster to adapt to the new situation.

EU initiatives such as the European maritime single window, which applies from 15 August 2025 [2], and the EU single-window environment for customs [3], which made its way into EU law in December 2022, will push digitalization in maritime industries, and, at the same time, these EU acts will increase the need for data and data exchange. The technologies of 5G and 6G fit naturally as enabling technologies: their wide fit-for-purpose radio spectrum and their enabling of customer-oriented features are the means to tailor these requirements. However, despite the recent advances in communication networks on land, offering dependable and high-speed data rates for marine communication remains challenging. 

The use of an air/space segment in mobile networks has recently gained a lot of attention as one of the solutions to tackling this challenge. For example, there have been recent works in the literature that have addressed this, mostly from a theoretical perspective. For example, in [4], unmanned aerial vehicles (UAV) were considered for the enhancement of the coverage of a hybrid satellite–terrestrial maritime communication network. This setup resulted in a difficult optimization problem, for which the authors proposed a method to solve it. Similarly, the authors of [5] studied the design of a space–air–sea non-terrestrial network (NTN) for energy-efficient resource allocation. Their goal was to maximize the system’s energy efficiency by collaboratively optimizing different parameters such as user equipment association, power control, and UAV deployment, which was also formulated as an optimization problem and solved in the paper.

From a more practically oriented perspective, the emergence and adoption of new production, propulsion and launching technologies for satellites has drastically reduced the deployment costs of space-based infrastructures and has made mega-constellations viable. Consequently, the use of satellite communication technology as a complement or even an inherent part of future network infrastructures is becoming increasingly interesting for various vertical markets and mobile network operators. The main standardization body for 5G/6G mobile networks, 3GPP, has already mandated in its Release 15 Service Requirements specification [6] that “The 5G system shall be able to provide services using satellite access”.

With Release 17, which was finalized by mid-2022, the integration of satellites/NTN into 5G mobile telecommunications systems has now been included in all the relevant specifications. According to the Rel. 17 Service Requirements [7], a 5G system with satellite access shall support:

- “Service continuity between 5G terrestrial access network and 5G satellite access networks”
- “Different configurations where the RAN is either a satellite next generation (NG-) RAN or a non-3GPP satellite access network, or both”, and also with satellites being part of the transport network.

Since the finalization of Release 17, equipment vendors have been quick to start ambitious development programs for suitable chipsets, components and units with remarkable success [8,9,10,11]. These advances will allow for rapidly bringing full 5G NTN implementations, providing direct access from the user equipment (UE) to satellites to the market with the promise to revolutionize connectivity for mobility applications. Already available today are 5G-compatible satellite backhauling solutions between larger vessels, aircraft or trains with their own 5G micro-cell or non-public network (NPN) and a 5G core.

Thus, it is clear that hybrid satellite–terrestrial communication networks will play, as expected, a pivotal role in providing connectivity in maritime environments. Key technologies, open issues, opportunities and challenges for these types of networks are described in [12,13].

Besides the coverage extension thanks to the aerial/space segment, another key technology that is paramount in providing coverage extension is multi-hop. The idea of multi-hop cellular networks gained a lot of attention for 4G networks, also known as IMT-advanced [14] networks. One of the most cited publications [15] dates back to 2004 and describes the related challenges for the technical and commercial success of multi-hop cellular networks using relays: the cost of a relay must be low enough to compensate for the loss of capacity resulting from dedicating part of the radio spectrum to a wireless backhaul link. To the best of our knowledge, no commercial relays are available on the market and logically, therefore, no commercial deployments exist. Instead, wireless backhauling in high-frequency bands [16], also known as millimeter wave (mmWave), has been widely deployed. It combines the advantage of high bandwidths and reduced deployment costs and effort, as no fibers connecting to the base station sites are required while not having to dedicate any spectrum for backhauling, as the cell operates in other, lower-frequency bands. Relay-like solutions regained momentum with 5G new radio (NR), as it introduced regular cell operation in mmWave high bands [17]. The corresponding solution is called integrated access backhaul (IAB), and it is motivated by applying existing mmWave spectrum licenses that are used for wireless backhauling to also be used for the actual cell (Uu-interface) or, alternatively, to also use the mmWave spectrum, which is mainly intended for the Uu-interface, for backhauling.

Additionally, besides hybrid satellite–terrestrial communication networks and technologies such as multi-hop, trends such as mobile edge computing/cloud (MEC) and NPNs, also known as private networks, have facilitated the research and commercial deployment of mobile radio networks with different geographical locations of network functions. In the context of MEC, which applies to many use cases [18], different splits are introduced to 4G and 5G networks, as described in [19]. This particularly includes the idea of having the core user plane locally, allowing for connecting MEC servers while keeping the control plane components of the core central. This principle was evolved for the 5G core, assuring a lightweight user plane that only consists of the user plane function (UPF). For example, the 5G cross-border service continuity project 5GCroCo discussed different split options and their implications in Deliverable D3.3 [20]. The typical motivation behind MEC is to enable deploying edge servers close to already-existing base station sites. NPNs typically aim at deploying networks on premises while keeping costs and complexity reasonable, which requires selecting an appropriate option for the “split” between the RAN and the core and between the core user and control planes. This can include the option of reusing the existing base stations of the public network to create public network integrated NPNs (PNI-NPNs), as discussed with an economic focus in [21]. A comprehensive discussion of different core split options is provided in [22]. 

Many, but not all, findings for wireless access backhauls and split options are relevant for the maritime scenario evaluated in this paper. Inspired by these related works, Section 3 contains an analysis of the applicable options for multi-hop maritime scenarios.

None of the works cited previously included aspects related to the deployment, testing and trialing of actual mobile networks endowed with a satellite component and multi-hop technology in maritime environments, which was the focus of this paper and one of the main goals of the 5G-ROUTES project.

The 5G-ROUTES project is funded by the European Union’s Horizon 2020 research and innovation program under the auspices of the European 5G Action Plan [23]. The European 5G Action Plan addresses the need for high-data-rate connectivity everywhere and all the time with a specific focus on the mobility vertical, encompassing all modes of transportation—land (both road and rail) and, especially important for this paper, sea or in the motorways of the sea.

The overall objective of the 5G-ROUTES project is to conduct advanced field trials of some representative and innovative connected and automated mobility (CAM) applications, seamlessly functioning across a designated 5G cross-border corridor (‘Via Baltica-North’), traversing Finland, Estonia and Latvia, in order to validate features and 3GPP specifications under realistic conditions so as to accelerate the widespread deployment of 5G end-to-end interoperable CAM ecosystems and services in digitized motorways, railways and shipways throughout Europe. For this purpose, a number of use cases have been identified, some of which address multi-modal transport, and these scenarios considered for trialing are described in the next paragraph.

Among the use cases defined in the 5G-ROUTES project in [24], four specific use cases focus on the quality and coverage of connectivity services. Two of these use cases focus on passenger transport services, one focuses on supply chain and freight transparency in multimodal transportation, and one focuses on smart ferries/vessels themselves. The aim of these different use cases is to study and pilot uses cases targeted for different customer groups in maritime transportation. The target is also to combine road and maritime transportation and to aim for continuous multimodal service, tracking and transparency availability. The use cases within the 5G-ROUTES project focus on two different themes. In the first theme, the use cases focus on network availability and coverage and how to improve the current services. The second theme focuses on network quality, e.g., latencies, throughput rates and download speeds.

To end this introduction section, we provide a summary of the main novel contributions of this paper and describe how it is structured in the different sections according to our proposed approach and methodology. The main contributions of this paper are:

- To conduct and present the results of a measurement campaign of 5G coverage at 3.5 GHz and 700 MHz to assess the (poor) mobile coverage in ferries for maritime scenarios.
- To propose different architectural solutions to provide a seamless 5G service connectivity to sea ferries operating across country borders, capitalizing on multi-hop and satellite technologies and to discuss on their pros and cons.
- To analyze and describe in detail the planned field trials that will take place to validate the architectural solutions, including selected KPIs, to quantitatively assess the performance of the proposed solution and the selected building blocks for the implementation of the end-to-end system.

In particular, the present paper is organized as follows. First, Section 2 provides a description of the maritime scenario considered in this paper and, based on the results of the measurement campaign on the maritime coverage referred to above, states the main problem at hand, i.e., that of providing seamless connectivity for maritime applications. Then, Section 3 discusses different architectural solutions to provide 5G connectivity in maritime environments. Section 4 then gives a description of the field trials that are, among others, planned on the ferry route(s) across the Baltic Sea between the ports of Helsinki/Vuosaari in Finland and Tallinn/Muuga in Estonia in the 5G-ROUTES project, focusing on the overall 5G trial architecture that integrates satellite and multi-hop connectivity. Finally, the paper concludes in Section 5 by summarizing the achieved findings and, also, providing a vision for applying the results of these investigations more generally to oceanic traffic.

## 2. Maritime Scenario and Problem Statement

On their journey across the Baltic Sea from Helsinki/Vuosaari to Tallinn/Muuga, ferries encounter significant variations in 4G and 5G coverage that prevent them from ensuring continuous onboard connectivity.

Close to land, the terrestrial base stations in the ports and along the coast are able to provide sufficient bandwidth. However, as is quantitatively assessed in the following, with increasing the distance to the shore, the signal strength decreases. Thus, as already discussed in the introduction, alternative connectivity solutions enabling better coverage and quality of service (QoS), such as satellites and/or multi-hop (vessel to vessel) relays, are considered in this paper.

**Figure 1:**
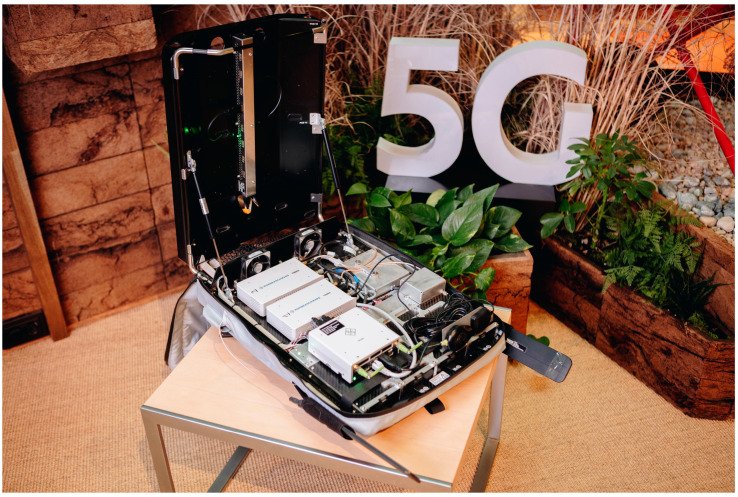
Equipment used for the received signal strength measurement campaign.

The following subsection presents the current 4G and 5G coverage status in the Baltic Sea. To perform the measurements, two cellular base stations currently deployed in the port of Vuosaari, operating at 3.5 GHz and 700 MHz, were used for the measurements. For the coverage measurements, based on the received signal strength at the end-device, a Rohde&Schwarz FR4 Freerider 4 backpack system was used, as shown in Figure 1. This equipment provides a complete and compact drive test system that can be temporarily installed inside a vehicle/vessel, significantly reducing the setup time for measurement campaigns.

### 2.1. Coverage Status in the Baltic Sea

**Figure 2:**
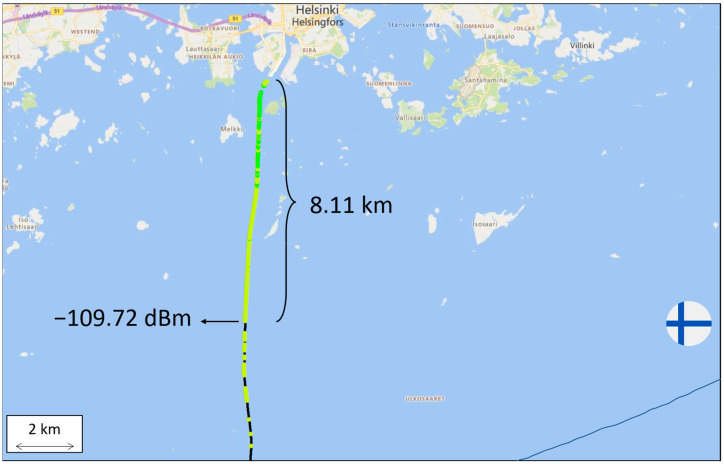
Signal strength at 3.5 GHz along the ferry route from Helsinki harbor. Greener color represents higher received signal strength and yellower color represents lower received signal strength. Black color represents power outages (i.e., received signal strength below −110 dBm.

Currently, a 5G base station operating at 3.5 GHz is located in the harbor of Helsinki, and the signal strength was measured along the ferry line that is depicted in Figure 2. Using the equipment shown in Figure 1, a set of received signal strength measurements were taken. The 5G signal reached 8.11 km from the shore with a strength of between −105 to −110 dBm and then deteriorated rapidly. The performance was better than initially expected, but not enough for covering shore to shore. A table listing the received signal strength values versus the distance can be found in Appendix A.

**Figure 3:**
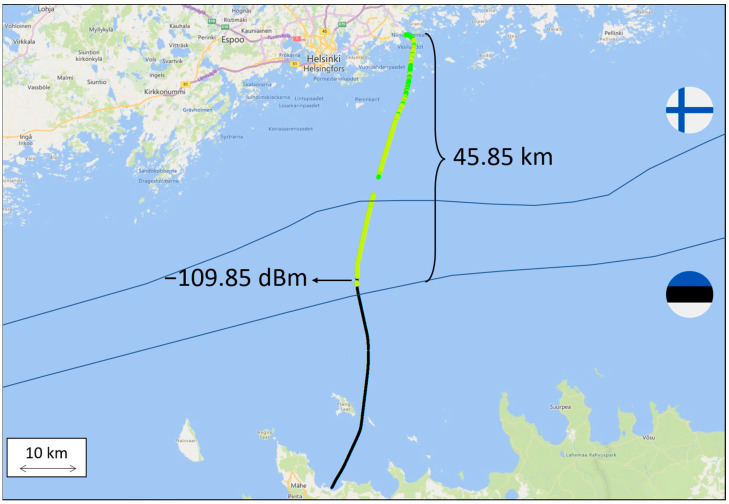
Signal strength at 700 MHz along the ferry route from Vuosaari harbor. Greener color represents higher received signal strength and yellower color represents lower received signal strength. Black color represents power outages (i.e., received signal strength below −110 dBm.

Similarly, a 4G base station operating at 700 MHz is located in the Finnish Vuosaari harbor and, in this case, the performed measurements indicated that the signal reached 45.85 km from the shore with a strength of between −105 to −110 dBm and then also deteriorated relatively quickly (see Figure 3). A table listing the received signal strength values versus the distance can be found in Appendix A. However, the signal could reach above 45 km even if using 4G; the technical limitation on 700 MHz 5G new radio (NR) is currently 35 km and, thus, covering shore to shore, even with the coastal base stations in Muuga (Estonia) and Vuosaari (Finland), is not possible.

From the measurement campaign conducted, a series of conclusions were obtained, as described in the two following sections.

### 2.2. Coverage Status in the 3.5 GHz Band

Using a 3.5 GHz carrier brings the operator multiple advantages and disadvantages. With regard to the advantages, we can name the following:

- The 3.5 GHz frequency band is well-suited for designing radios with phased-array antennas due to the wavelength characteristics of this frequency range [25]. With a shorter wavelength, it is easier to design radios with phased-array antennas and enable beamforming. This is because the size of the antenna elements in a phased array is directly proportional to the wavelength of the signal being transmitted or received. Higher-frequency signals, such as those in the 3.5 GHz band, can be accommodated with smaller and more closely packed antenna elements. This allows for the creation of a phased array with a larger number of elements, making it easier to enable beamforming and to leverage its technical advantages for optimal coverage and improved interference rejection along the ferry lines.
- More bandwidth is available to facilitate even the most challenging use cases in terms of capacity (e.g., dense harbor areas).
- High loss propagation allows for better control of the designated coverage with good flexibility for interference mitigation.
- The commonly used time-division duplex (TDD) technique allows for the flexible configuration of up-link (UL) and down-link (DL) frequency resources based on the service type. The management of interference in non-synchronized deployments between different parties is challenging in practice. However, recent 5G advancements, such as the ability to mute specific transmission intervals and coordinate transmission between network nodes, make the use of this flexibility more practical.

Regarding the drawbacks, the following are highlighted:

- Shorter wavelengths compared to low-frequency waves result in a reduced ability to penetrate through obstacles such as buildings, ships and other solid objects. This presents a challenge in terms of ensuring reliable coverage inside such environments.
- The high propagation loss of high frequencies is a significant drawback when the required service distances are considerable, as is the case in the analyzed ferry use case.
- Radio propagation is strongly impacted by heavy precipitation such as rain and snow, which are common in the region. These weather conditions can absorb, reflect or scatter the radio waves, leading to signal attenuation, interference and a decrease in the coverage area. As a result, the quality and reliability of the communication link may be degraded, making it challenging to maintain a stable and robust network performance.
- Commonly, the TDD spectrum usage technique is used in the given bandwidth, which imposes a challenge in terms of precise network synchronization and requires a common timing source for all the operating parties due to side harmonics. Otherwise, a significant guard band is needed between the operating frequencies.
- In addition, it is important to consider that the common TDD pattern required between different operating parties may limit the availability of services such as ultra-reliable and low-latency communications (URLLC). According to 3GPP TR 38.913 V17.0.0 (2022-03), the target for user plane latency in URLLC should be 0.5 ms for UL and 0.5 ms for DL.
- The prioritization of the DL transmission in the most commonly used TDD patterns can result in limitations to the UL transmission, leading to a negative impact on UL-intensive services.
- Finally, if there is a prolonged lack of a synchronization reference in TDD networks, there is a high risk that the network will be disabled. Robustness against this situation can be obtained by means of having multiple synchronization sources for redundancy: GPS, PTP via a transport network, operating local high-precision clocks, etc. Another solution for operating in non-synced environments is operating with extended guard bands. However, this would imply a significant reduction in usable bandwidth.
- Some geographical areas have restrictions to using the 3.5 GHz band due to other equipment being operated on the same frequency.

### 2.3. Status in the 700 MHz Band

The main advantages of using a carrier frequency at 700 MHz are:

- A low loss propagation and, thus, covering long distances requires less transmitted power [26].
- In the 700 MHz band, the frequency-division duplex (FDD) spectrum usage technique is mainly implemented in today’s networks. Accordingly, operating at this band does not impose any synchronization challenges and, thanks to the fact that DL and UL slots are always immediately available, ultra-low latency use cases can be better supported.
- A reduced power consumption of user equipment, leading to it being more suitable for battery-powered devices and IoT.

Concerning the disadvantages of 700 MHz operation, we can name the following:

- The available bandwidth is limited in the 700 MHz band.
- UL and DL bandwidths are immutable; thus, there is no flexibility for dynamic bandwidth management, which may be important in certain variable-rate applications.
- The low frequency of operation poses certain hardware design challenges, especially related to the size of certain components which, in particular, makes the implementation of beamforming rather inefficient.
- The low propagation losses, strong diffraction and strong reflection properties of the 700 MHz band present challenges in terms of controlling coverage areas, potentially leading to interference, particularly in regions where other technologies might be still using this spectrum.

### 2.4. Problem Statement and Future Vision

Besides the details provided in the two subsections above, there are several aspects that need to be considered when planning and using radio access networks and/or radio links over open seas and on moving ships. As already pointed out, different weather conditions—fog, waves, ice, etc.—can affect radio waves to a large extent, which makes the coverage conditions inconsistent [27]. The greater the involved distances, the greater the potential negative performance impact. Such changing channel conditions not only impact the signal but also the interference power strength variability, which can be especially challenging at low frequencies as they propagate larger distances. Moreover, on open seas and border areas, different legislation (e.g., frequency regulations on international waters) and frequency coordination agreements need to be taken into account.

**Figure 4:**
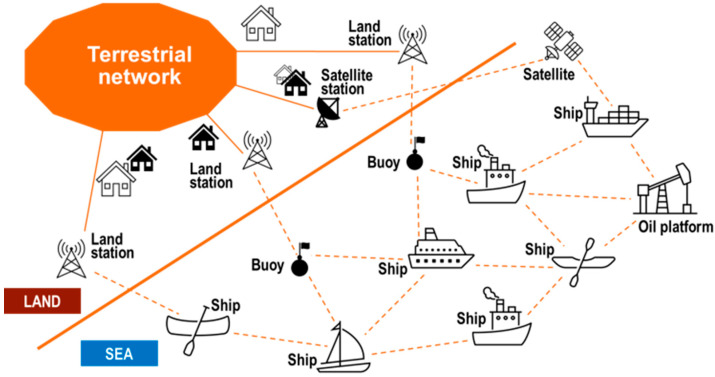
Vision for the high-level solution of the seamless connectivity problem in maritime environments.

To address the technical aspects listed above, our 5G connectivity vision for the maritime ecosystem consists of three main components to provide seamless connectivity: short distances from land stations, and a combination of multi-hop and 5G non-terrestrial networks to cover mid-distances up to international waters. A graphical schematic of this vision is provided in Figure 4. Additionally, multi-hop [28], satellite connectivity and their combination [29] will improve not only the maritime scenario but also, e.g., aviation environments, where they will complement terrestrial 5G networks and bridge coverage gaps in rural and remote areas. Regarding other areas that can benefit from satellite connectivity, the 5G Automotive Association has partnered with the European Space Agency to investigate the use of satellites [30], recognizing the continuous and ubiquitous connectivity essential for many automotive use cases, albeit with a need to improve the technological maturity and cost-effectiveness of the solution.

## 3. Maritime Network Architecture and Solutions for Seamless Connectivity

As has been discussed previously, it is important that the vessels are always (best) connected. The need for communication and connectivity increases when a vessel is operating on more crowded or more challenging waters. When a vessel goes to open waters, the requirements for connectivity might decrease. This also sets the scene for maritime clusters, where one solution does not fulfill all the requirements, or it might be too sophisticated or expensive for some cases, while in some environments, it could be a minimum requirement in the future. Hence, in this paper, we studied several different connectivity scenarios, which might supplement each other with the vision presented in the previous section, according to which 5G will become the primary technology for seamless connectivity in the maritime industry in the near future. Terrestrial network connectivity is already used in ports and on ships close to the coast, but in order to ensure seamless connectivity and real-time data exchange, it is necessary to develop the provision of 5G connectivity between ships by creating a 5G multi-hop network, as well as to ensure connectivity with 5G non-terrestrial connectivity in offshore waters. Coverage with 5G is only one aspect of deploying a 5G network at sea level, where the most important thing is to ensure the QoS required for each use case (usually measured via more fundamental KPIs such as latency, throughput, availability, etc.).

**Figure 5:**
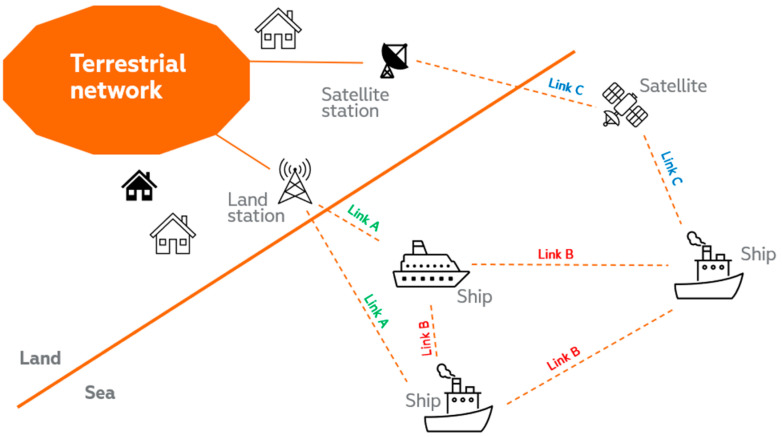
Main links to be studied for the seamless connectivity problem in maritime environments.

In the following, we will separately discuss the relevant architectures for the maritime multi-hop connectivity scenario. For this purpose, it will be useful to define three types of links (Link A, B and C) among the different elements that are depicted in Figure 4, depending on the geographical nature of the involved communication pairs (sea, land, air/space). Link A covers land (sea communication), Link B addresses sea (sea communication) and Link C relates to communications with the presence of an air/space segment (see Figure 5).

We first discuss the possible architectural splits between the components on the ships (onboard) and those not on the ships (onshore) related to Link A. Within this discussion, we assume IP network connectivity between the components without discussing how it is realized (all nodes, also known as network functions, forming a mobile radio network are interconnected over internet protocol (IP) connections. This also applies for the interfaces between the RANand the core. Theoretically, any internet connection with endpoints at arbitrary geographic distances could be used, but practically, the requirements regarding latency, reliability, throughput and security need to be fulfilled.). The second discussion focuses on possible realizations of this IP connectivity, starting from single-hop ones and then considering multi-hop, covering Links B and C. Finally, for the case that IP connectivity is realized through an onshore mobile radio network, we discuss how to achieve uninterrupted service delivery if ships operate between the shores of different countries and where those countries are served by different mobile network operators (MNOs).

### 3.1. Network Splits for Coast-to-Vessel Communication 

**Figure 6:**
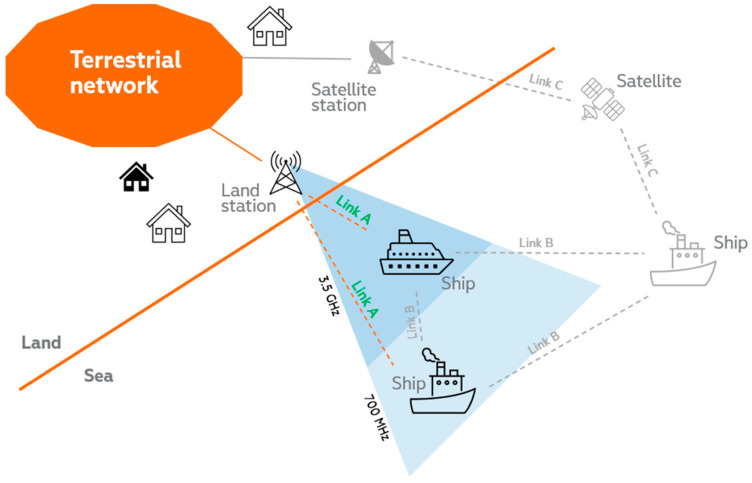
Link from the shore to the sea—Link A.

The focus of this section is to provide different architectural alternatives of network splits to address the connectivity from the coast/shore to the vessel/onboard. A general depiction is provided in Figure 6.

**Figure 7:**
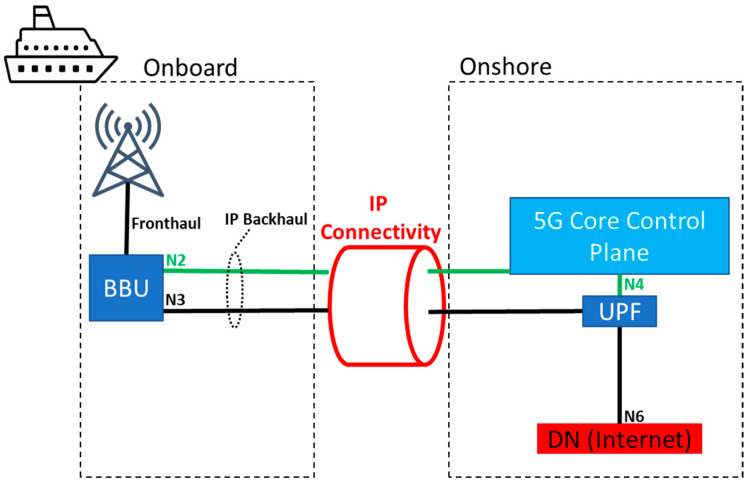
Core onshore (also known as) wireless access backhaul.

The first possibility, shown in Figure 7, corresponds to what is commonly known as wireless access backhaul. In this case, the base band unit (BBU) would be located onboard, and the N2 and N3 interfaces that connect it to the 5G core control plane and the data plane onshore would be realized via IP connectivity (as pointed out above, the discussion on the architecture is agnostic in terms of the underlying IP technology available, as long as it meets certain requirements) between ship and shore. In this case, access to further data networks (DNs) (e.g., the Internet) would be carried out onshore via the N6 interface. A loss of IP connectivity would almost instantly lead to a total network outage due to timeouts on the control plane on the N2 interface. Even before that, application services on the user plane would likely stop working as backend servers in the DN would not be reachable. In terms of infrastructure costs, this split would only require a BBU and onboard antennas.

**Figure 8:**
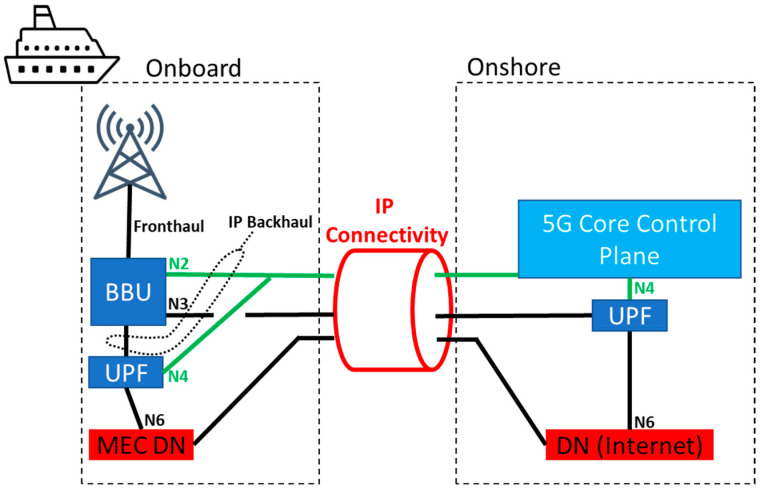
Control plane core onshore.

The second considered architectural split is depicted in Figure 8, where only the control plane functionalities of the 5G core remain onshore, and the data plane is moved to the vessel premises. This architecture enables the possibility of accessing an additional DN that physically resides in the vessel as well. The most straightforward application would be accessing MEC servers to provide services for users onboard. Application services running the backend on an MEC server might for a very short time, usually few seconds, survive an IP connectivity outage. After that, control plane timeouts would cause a total network outage where all the connected end-devices would go into a “no service” state. In terms of infrastructure costs, this split would require a BBU and onboard antennas together with servers that host the UPF and MEC functionalities.

**Figure 9:**
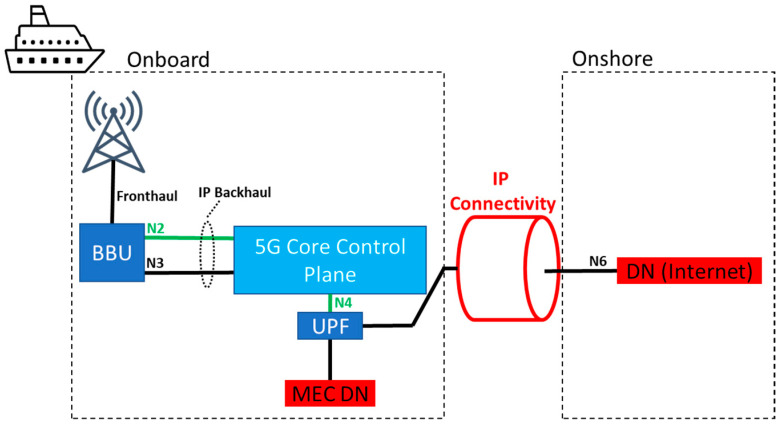
Core onboard.

Finally, the third considered option is depicted in Figure 9 and consists of having the full 5G network (RAN and 5G core) onboard. Only connectivity to external DNs, such as the internet, would be achieved via IP connectivity. A loss of this connectivity would only cause internet services to fail, while all MEC-hosted services would remain operational. However, many modern systems use public cloud-based management and monitoring platforms that would also stop working upon a loss of IP connectivity. This would affect the network and end-devices such as routers in cases where they are cloud-managed, but usually this would not lead to a complete application service outage. In terms of infrastructure costs, this split would require a BBU and onboard antennas together with servers that host the whole core and MEC functionalities. Accordingly, when considering the cost scaling with the number of vessels, this last architectural split is the one that would require the highest infrastructure costs, and the wireless backhaul would be the one with the lowest infrastructure costs.

Table 1 below summarizes the main differences among the three splits described above.

### 3.2. Single- and Multi-Hop Ship–Shore Connectivity

As has been discussed previously, the 5G multi-hop method offers an interesting option to extend the coverage on the sea. With the available information about the real-time vessel movements and historical data, a multi-hop network could be planned for vessels so that it would gather the most important maritime corridors by utilizing operative vessels as moving base stations or multi-hop nodes. Such a solution could save infrastructure investments and enable more flexible communication for maritime scenarios. From a risk and resilience analysis point of view, such an option could also be interesting due to the freedom from infrastructure built onshore. For example, in emergency situations, multi-hop node vessels could provide fast and agile support for rescue teams and operations by sharing their connectivity capacity.

By creating a “dynamic” 5G mesh network in the corridors of intensive vessel traffic, it is possible to provide backup channels for ensuring 5G connectivity. In cases where one connection channel is lost, a vessel could connect to another vessel without losing connectivity. We assume that, with such approach, 5G could be more reliable compared with the existing solutions.

**Figure 10:**
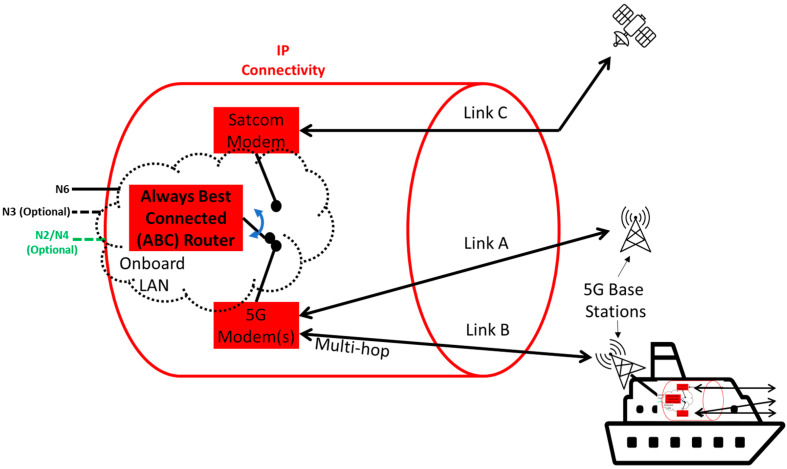
IP connectivity with always best connected (ABC) router.

Figure 10 depicts different options to achieve IP connectivity for the onboard–onshore splits discussed in the previous section. One or more always best connected (ABC) router(s) are the central elements for enabling flexible and resilient connectivity between onboard and onshore components. A local area network (LAN) is assumed on the ship, which also includes the special case of the LAN only consisting of an ABC router. The LAN transports at least the N6 interface to the onboard network. Optionally, depending on the split option discussed in the previous section, N2, N3 and/or N4 interfaces could also be provided. The wide area network (WAN) interface of the ABC router could select from among different options, namely satcom or 5G (incl. a fallback to earlier cellular network generations). If capable, the ABC router could also aggregate several connectivity options and also have more than one modem of each type. It is also not precluded that one or more of the modems depicted separately in Figure 10, e.g., the 5G one, would be integrated in the ABC router. 

For the use of satellite communication in conjunction with 5G, different architecture options exist, as described in the introduction. Within the implementation options described by ETSI [31], the following two were identified as suitable for the maritime scenario evaluated within the trials described in Section 4:

- ETSI Scenario A4: indirect access via an NTN transport network.
- ETSI Scenario A5: indirect untrusted access using non-3GPP interworking functionality (N3IWF) with a secure entity (SER) between the micro cell and the NTN terminal to be integrated into the 5G core network.

**Figure 11:**
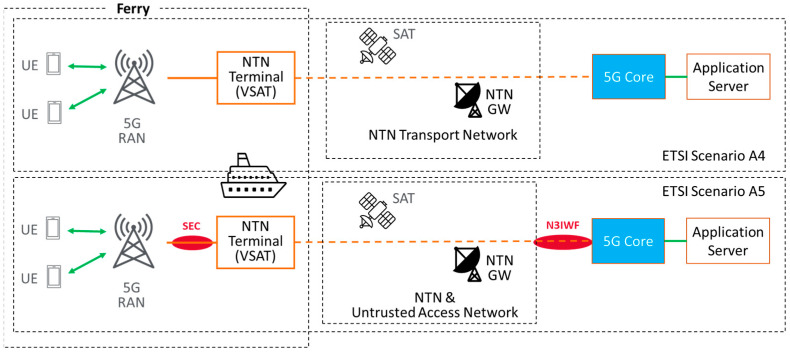
Options for realizing satellite connectivity for ferry trials.

A block diagram for both options, A4 and A5, employing state-of-the-art very-small-aperture terminals (VSAT) is depicted in Figure 11.

Due to the ease of establishing seamless integration with the 5G core network (without the need to implement the N3IWF functionality), the first option (scenario A4) is currently being implemented as a baseline, as will be described in Section 4.3. Option A5 with N3IWF would provide further convenience regarding access authentication, which is not subject to technical performance evaluations but could be theoretically evaluated.

For a 5G modem, the special case of multi-hop connectivity is also shown in Figure 10. In that case, an onboard 5G network on another ship, following any of the splits presented in Figure 7, Figure 8 and Figure 9 in the previous section, could provide connectivity to the considered ship. The other ship would also have an ABC router, enabling different options to connect to yet another ship or an onshore network. In the case of only one 5G modem with one SIM, this is non-trivial, as the SIM would have to be admitted to the 5G network of another ship and different onshore networks. The corresponding solutions heavily depend on which split option has been selected. In the case where multiple 5G modems are available, one could be used for ship-to-ship multi-hop connectivity while the other one could be used for ship-to-shore connectivity. The ship-to-ship 5G modem would have to be configured to prevent it from connecting to the 5G network on its own ship. The ship-to-shore 5G modem should benefit from seamless cross-border/-MNO service continuity, as discussed in the next section.

### 3.3. Cross-Border/-MNO Service Continuity for Ship–Shore Connection

Cross-border/-MNO service continuity for 5G networks has been studied in the EU-funded projects 5GCroCo, 5G-MOBIX and 5GCARMEN. While nuances and naming might be different across the projects, three solutions have been proposed:

- Inter-PLMN handover;
- Release with redirect (RwR);
  ⚬ With S10/N14 interface;
  ⚬ Without S10/N14 interface;
- Modem-based solutions;
  ⚬ With one modem;
  ⚬ With two modems.

The first two, including the two options for RwR, are described in [20], and their performance, regarding their service interruption duration, has been evaluated in [32]. Inter-PLMN handover results in a service interruption time of around 100 ms, which is typically not noticed by application services. RwR with an S10 interface (5G non-standalone (NSA)) or an N14 interface (5G standalone (SA)) results in an interruption time of less than one second, which can be noticeable but likely with no serious impact for most of the use cases evaluated within the 5GROUTES project. Without an S10/N14 interface, it depends on the time required to notice a loss of 5G network connectivity and reconnecting. This is typically in the range of one to a few seconds.

Regarding the third option, cross-border/-MNO service continuity by actively adjusting the parameters of one or two 5G modems is discussed in [33]. With only one modem available, the current network connection must first be terminated, and then a manual network scan must be performed. Limiting the scan to certain known networks and/or frequency bands can speed up the process, but it usually results in multiple seconds of service interruption. In the case of having two 5G modems, such an interruption would be prevented, as one modem would remain connected to the current network while the other one would be set to search for the new one and connect to it. This solution is not very attractive for automotive scenarios, which were the focus of the three mentioned projects, as each modem significantly contributes to the cost of the overall solution, and it would be unclear which operators and corresponding SIMs to choose. In case of sea ferries, this option could make more sense, as the cost of another 5G modem is only a small portion of the overall cost. ABC routers often include more two or more 5G modems. Furthermore, it would be clear which operators and SIMs to choose, as these would correspond to the countries connected by the ferry line.

Note that this only affects the IP connectivity discussed in previous sections. This is used to transmit data according to the selected split between the onboard and onshore networks. The actual network on the ship could, but does not have to, be completely different than the ones providing ship-to-shore IP connectivity. 

## 4. Trials in the Baltic Sea Corridor (Tallinn–Helsinki)

In order to practically validate the architectural solutions and strategies proposed in the previous section, trials are planned in the Muuga/Tallinn–Vuosaari/Helsinki corridor in the context of the 5G-ROUTES project. The plan is to utilize Eckerö Line’s Finbo vessel as a test platform and to equip it with the necessary radio and communication devices. The plan also includes supplementing Finbo with a second vessel/boat, which can operate more freely, since Finbo operates between Vuosaari (Finland) and Muuga (Estonia) according to a tight daily schedule. This section provides a description of the planned trials, for which a set of use cases have been defined.

As has been described in the previous sections, the maritime use cases will utilize 700 MHz, 3.5 GHz and satellite communication as the backbone and ferry coverage. These scenarios will be extended with the multi-hop connections. All these solutions will be tested and analyzed both separately and together in a real maritime operation environment. The 5G-ROUTES use cases were defined so that they will cover most of the potential customer and user groups in the ferry, i.e., passengers, freight companies and ferry operators/staff and, hence, the future opportunities and benefits for these user groups could be analyzed. In the maritime pilots, which will be prepared and pre-tested in lab trials that are starting in the first half of 2023, satellite communication will be used to provide an alternative for cellular-based backbone connectivity. It is clear that, especially on open-sea cases such as the one considered in this paper, the role of satellite communication becomes paramount, while closer to shore, other options might be more suitable. Accordingly, studying strategies for load balancing between cellular and satellite backbone connection will be part of the trials.

### 4.1. Description of 5G-ROUTES Use Cases

The 5G-ROUTES project will trial four different maritime use cases, which will focus on the quality and coverage of connectivity services at the sea. The corridor between Finland and Estonia is used for piloting and serves as example for other maritime environments. It offers a fruitful piloting environment, since it has a lot of vessel traffic and it provides a cross-border transport corridor, which also belongs to the TEN-T core network [34].

Use cases #1 and #2 will focus on passenger services. Use case #1 will include a sophisticated mobile game, which is built on augmented reality. In the game, users will share a collaborative gaming experience via their mobile phones, i.e., the devices will be connected to a game server deployed on MEC hosts close to the 5G core. The devices will continuously share their locations, orientation and interactions with other players. At the same time, the server will process the players’ data in real time and respond back to the devices with information to compose the game scene.

Use case #2 will focus on a virtual reality application, where the users will use a VR headset and a laptop connected to the 5G network to participate in an immersive meeting session onboard a ferry. In a different location, the presenter will be captured in front of a green screen and embedded in a virtual environment, which will be streamed to a multicast streaming server deployed in the core/edge. On the ferry, the audience will see the VR stream (4K/8K video data) and will be allowed to interact with the details in the immersive scene. This node will distribute the VR stream to the audience on the ship. The viewers will be able to point out details of the immersive scene and send voice messages back to the presenter. 

Use case #3 will focus on freight transportation and the continuous tracking of cargo. The aim is to drive a test vehicle inside the ferry and attach the vehicle’s OBU and IoT sensors to the 5G network. While the ferry is moving, the connection between the IoT sensors, the OBU and the backend systems will be analyzed. Real-time kinematic (RTK) location data sent from a server in the shore and temperature data will be used as sample data to demonstrate the data flow between the devices and the backend systems. Massive machine-type communication (mMTC) will be piloted and tested under this use case. For these tests, an Android application has been developed, which runs on several mobile phones (at the time of writing, almost no smartphone allows attaching to 5G SA private networks. Smartphone vendors use whitelisting, allowing phones to attach to certain networks, usually from main network operators. Thus, the Android application will most likely have to run inside Windows BlueStacks Android emulator.) at the same time. Each of these devices will open and maintain thousands of TCP data streams and send status messages from the devices over each stream every 1 to 60 s. With this mMTC pilot, the aim is to demonstrate the use case where various devices and units are simultaneously and continuously connected to the same 5G base station. This could, for example, happen in ports, when freight vessels carrying containers and trailers are approaching the dock and where other freight vessels are waiting for their transportation.

Use case #4 will focus on smart ferry applications. The main goal of this use case is to utilize IoT connectivity and equip the vessel with several IoT sensors. These sensors will report continuously about the vessel’s situational awareness information, such as temperature, air quality, humidity, etc. Such data can be used to obtain a general overview of the conditions of the ferry or to monitor safety- and security-related issues, which are more critical to vessel operators and staff. It is different than use case #3, as it will consider the ferry itself as opposed to the goods and vehicles the ferry transports. 

The use cases described above represent only a part of future maritime CAM services, but they highlight different user categories and customer groups of the services. Common to all these use cases and pilots is that during the trials, the real time data interchange between server and on ferry devices will be recorded and analyzed, allowing for studying QoS, coverage and QoE. 

In the planned trials, the route for the maritime transportation is expected to be the link between Helsinki/Finland and Tallinn/Estonia. The planned connectivity trials will be implemented on Eckerö Line’s Finbo ferry, which will be equipped to act as a test platform while travelling between the ports of Vuosaari and Muuga across the Baltic Sea.

### 4.2. Overview of Trial Architecture

**Figure 12:**
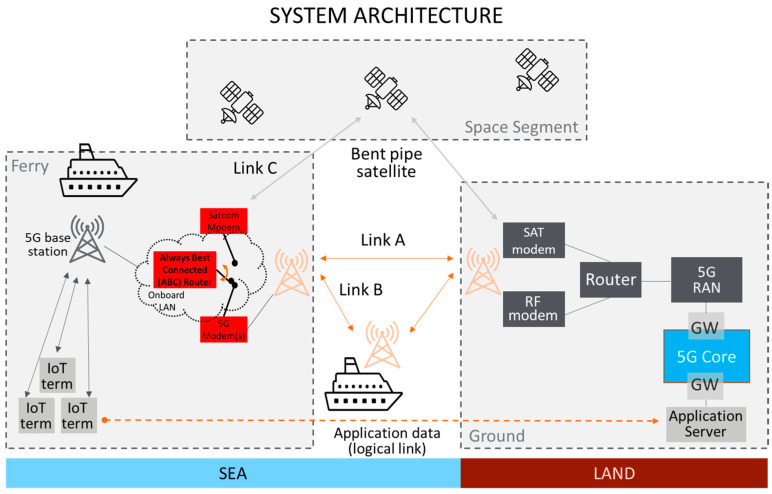
Overview of the trial architecture.

The architecture of the ferry trials is displayed in Figure 12. The installed infrastructure consists of a number of onshore 5G base stations in the port of Muuga and onboard 5G systems. This could allow evaluations in the port areas and on the ferry route in-between to validate the use cases related to multimodality, goods tracking and providing uninterrupted connectivity from Tallinn to Helsinki.

Out of the 5G network split options discussed in Section 3.1, the third one has been selected for the trials, i.e., where a self-confined 5G network is deployed onboard. This is out of practical considerations, as a stable and performant ship-to-shore connection is part of the research and evaluation tasks within the 5G-ROUTES project. Having the core control plane or the whole core onshore can lead to the failure of the entire network, making trials focusing on user plane performance difficult. From an economic perspective, it makes sense to minimize the number of components on the ship while still keeping the option of deploying backend applications to MEC hosts. This corresponds to the second split option, where a centralized control plane core is deployed onshore with a decentralized user space core (UPF) onboard for MEC access. By deploying the whole core onboard for trials, the project will also be able to finally answer the question if, and under which circumstances, it would be possible to use an onshore, centralized, control plane core.

The ABC router will be realized through a 5G router that will also allow for connecting a VSAT modem over ethernet. It is envisioned to also evaluate ABC routers capable of using two SIM cards in parallel (dual-SIM-dual-active). For the satellite communication, backhauling option A4 (see Section 3.1) will be used, which is fully sufficient for the trials and does not require the deployment of an N3IWF.

With this setup, the performance of all single-hop (called Link A and Link C in Section 3) and multi-hop (Link B) connectivity options could be evaluated. Seamless switching between links will be carried out by an ABC router, and aggregating multiple links is not within the main scope of the trials. The rationale behind this is that, among the different parts of the journey, different links are preferred, as discussed in Section 1, Section 2 and Section 3, and it is, if at all, potentially beneficial to aggregate multiple links only during very short time period.

For the time being, it is not clear which option(s) for cross-border/-MNO service continuity will be deployed in the onshore networks. Initial trials will therefore use modem-based solutions not requiring network-side support.

### 4.3. Description of Planned Satellite Connectivity Trials

Currently, at the time of writing, the set-up for the implementation of satellite access on the ferry as a transport network is being prepared. It basically consists of an integrated satellite antenna/modem assembly that needs to be connected to the ferry power supply and via ethernet to the (backhaul) router of the ferry’s 5G cell. This assembly will be installed on the upper deck of the ferry with the Ku-band antenna pointing towards zenith—one of the terminal options currently being considered is the Kymeta Hawk u8 [35]. The satellite link is planned to be provided by the OneWeb constellation with the access link from the ferry to the satellite in Ku-Band (at 10.7–12.7 GHz for DL and 14.0–14.5 GHz for UL) and the feeder link from the satellite to the gateway—that then seamlessly interfaces with the onshore 5G core network—in Ka-Band (at 17.8–20.2 GHz for DL and 27.5–30.0 GHz for UL). 

As described in general in the section below, the planned testing campaign will initially measure the performance of the satellite link—i.e., throughput, latency and availability—in a standalone mode. Then, the final field trials will assess the overall connectivity performance for the entire ferry trip based on the above KPIs. Based on these results, recommendations for a prototypical and ultimately full operational implementation will be derived.

### 4.4. Description of Planned Coastal 5G Connectivity Trials

A trial plan for testing the maritime solution involves several key steps to ensure the success of the implementation. Firstly, the installation and integration of a private network core and radio base stations on Eckerö Line’s Finbo ferry will be performed. Secondly, a controlled environment test will be carried out to verify that the private network core and radio base stations are working correctly. Next, a series of tests will be conducted on the ferry to evaluate the network’s performance and identify any potential improvements or issues.

The tests will be started by testing one frequency and connectivity solution first. This functional testing will be repeated for each solution, and when all solutions have been proven to work as planned, the next phase of testing will be started. During the next phase, the different solutions will be tested together and will focus on the balancing between the solutions. This dual testing will be repeated for the different solution scenarios. When all the scenarios have been proven to work, then the final phase of tests can be started. The third phase of testing will cover all the connectivity solutions, and the aim is to find the best overall solution to fulfill the demand of continuous and high-capacity connectivity solutions for maritime environments. 

Simulations of the multi-hop connectivity will be performed by setting up a number of vessels as moving base stations or multi-hop nodes in the maritime corridor. During normal operation, the network will be monitored to ensure stable and reliable coverage. The network’s ability to maintain 5G coverage and local services in the event of a lost shore link will also be tested, simulating scenarios such as search and rescue operations, disaster relief or armed conflicts.

Key performance indicators such as latency, throughput and reliability will be measured and analyzed. Based on the results, any necessary changes will be made, and additional testing will be conducted to verify the effectiveness of these changes. Data will be collected throughout the testing process to inform future developments and scaling decisions.

Finally, the results of the trial will be compared with existing solutions to evaluate the benefits and limitations of the multi-hop approach. The findings will be shared with relevant stakeholders to guide future developments.

### 4.5. Performance Measurements

The main purpose of the performance evaluation is to assess how well the network and the use cases developed in the 5G-ROUTES project fulfill the requirements. Starting from the requirements of the use cases, as described in Section 4.1, a set of target KPIs were defined related to latency, throughput, coverage, reliability and position accuracy. The KPIs are aligned with the KPIs identified by the 5G PPP [36]. The network KPIs address the performance and capability of the network, such as the peak data rate and user plane latency and are measured using standardized procedures using synthetic traffic [37]. A tool called tenant web portal has been developed, acting as a data repository for the measurements and as a visualization tool for the KPIs.

The use-case-specific KPIs will be measured within trials from the first half of 2023 until spring 2024. Test scenarios were defined to measure the data needed for the KPIs during ferry operation, including cross-border/-MNO service continuity. After the tests, the data will be analyzed, and KPIs will be calculated and visualized. Starting from the results of the different test scenarios, conclusions will be drawn on the performance of the network and the use cases. At least two iterations will be applied within the trials for the possibility of performing actions to improve the performance of the network and use cases.

### 4.6. Vision of Multi-Hop and Trial Possibilities

**Figure 13:**
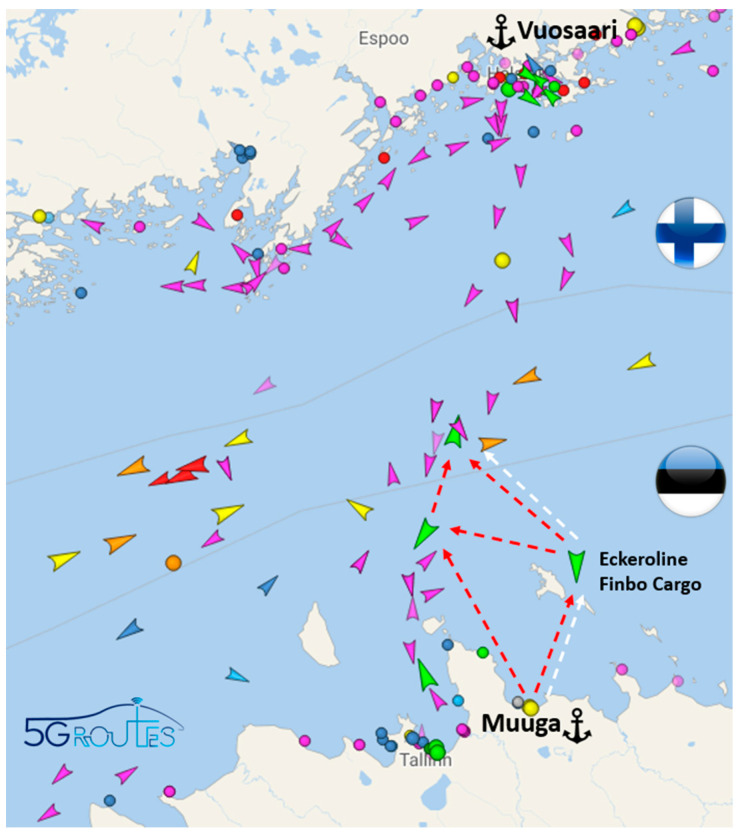
Vision of multi-hop trials.

The overall vision presented in this paper for the maritime scenario will be validated through demonstrations that include multi-hop (see Figure 13). These demonstrations will show how to ensure the selection of several possible connections based on the best QoS. The trials will be performed in two phases:

1. A vessel connected with only one route from the vessel where the 5G network is deployed;
2. A vessel connected with redundant routes from several vessels with 5G networks deployed.

**Figure 14:**
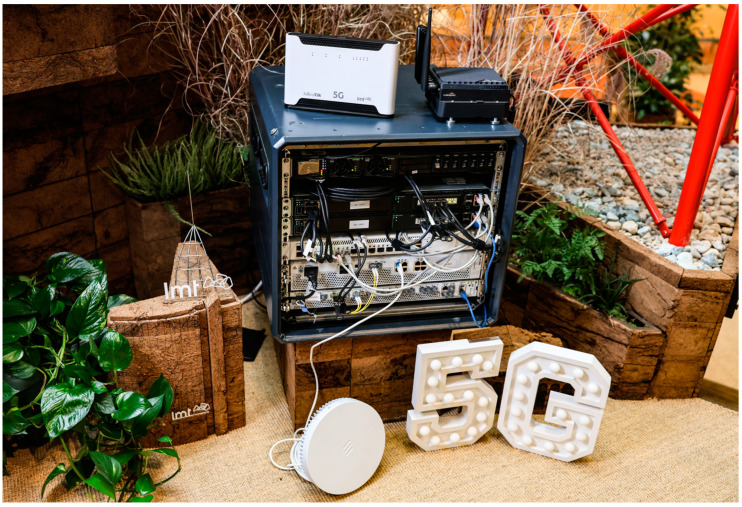
The 5G private network core and baseband unit.

The planned trials have similarities with the IAB concept [38,39], but considering that the 5G IAB functionality will not be available during the project’s lifetime, the implementation and use of NPN networks on two ships were chosen as the primary solutions. To prepare for the first phase of the connectivity trials, the 5G private network provided by Ericsson has been deployed in a test lab located in Latvia, as it can be seen in Figure 14. The solution provides all the necessary 5G functionality, and it is very compact and quick to deploy, and it consists of the following main components:

- Two DELL VEP4600 network controllers;
- A 6651 baseband for RAN;
- A 6250 power unit for the baseband;
- An AIR 6449 outdoor antenna operating in the 3.5 GHz frequency;
- A GNSS receiver and GPS antenna;
- Cloud management;
- Centralized extended network management.

In multi-connectivity, redundant routes can either be used as a backup or for load-balancing scenarios. Multi-hop backhauling for range extension is mainly considered with mmWave frequencies, but in the planned trials, a spectrum below 6 GHz will be used as a primary alternative because higher frequencies in the millimeter domain are expected to have several disadvantages in providing seamless cross-border connectivity. 

In a multi-hop network, the increased number of hops, the distance between vessels, the used frequency for backhaul, the limited nodes resources due to congestion and other aspects have a clear impact on the quality of service (QoS). Accordingly, in the planned trials, measurements of the latency, throughput and signal-to-noise ratio will be carried out to identify and validate the assumptions about the factors affecting the provided QoS.

The time frame of the execution of the 5G-ROUTES project will not allow for sufficient studying of the future standard implementations of Release 17 [39], but it will give a benchmark for future extended 5G coverage trials in measurements of hybrid backhaul solutions with current cellular and satellite technologies. Nevertheless, the need for improved coverage and cellular 5G service will be verified by the piloting ferry operator and the 5G-ROUTES use cases.

## 5. Discussion, Conclusions and Future Vision

This paper described auspicious concepts—cellular backhauling via coastal stations, satellite connectivity and multi-hopping—for providing continuous 5G connectivity in maritime scenarios in general and, in particular, in the ferry route from Helsinki to Tallinn, which will be investigated and trialed in the 5G-ROUTES project. The results of these analyses and field trials will be available towards the end of the project in the 2024 timeframe and will give the following clear indications on the logical steps towards prototypical and ultimately full operational implementation:

- The necessary technical developments and adaptations;
- The maturation of the value chain and service exploitation models;
- Inputs to the evolution of future 3GPP releases (specifically with IAB and NTN).

There is significant potential to build upon the findings of the 5G-ROUTES project to address more general maritime scenarios—such as longer ferry routes, for instance, from Tallinn to Stockholm—or even open-sea use cases. Multi-hop mesh architectures (see Figure 4) are emerging that use a variety of different elements such as base and relay stations on islands, on ships, on oil platforms and even smaller vessels, on airborne platforms and combined with satellites to connect to a 5G core network.

The future vision of the 5G connectivity maritime ecosystem involves three main scenarios to provide seamless connectivity:

- Short range—from land stations;
- Multi-hop as a mid-range solution for maritime corridors and internal waters;
- 5G NTN to cover international waters.

These three scenarios will enable a multitude of additional use cases, supporting enhanced situational awareness and improved goods tracking, improved ship-to-ship and port-to-ship connectivity and also autonomous/automated vessels, making traffic, travel and transport on the oceans safer and more efficient.

In addition, road and rail transport will also benefit from multi-hop and satellite connectivity as well as their combination, which could complement terrestrial 5G networks and help in closing coverage gaps in rural and remote areas. Continuous and ubiquitous connectivity is beneficial to and even required by many automotive use cases even though the technological maturity and cost-efficiency of the proposed solution both need to be further improved. 

The concepts developed in the 5G-ROUTES project and the results of the field trials will contribute to the vision of continuous 5G connectivity—not just along ferry lines across the Baltic Sea but ultimately everywhere and all the time.

## Figures and Tables

**Table 1:** Summary of the main differences among the three different network splits.

Split	BBU	Core (UP)	Core (CP)	MEC	IP Failure	Cost
Wireless backhaul (Figure 7)	Onboard	Onshore	Onshore	No	Immediate network outage	Lowest
CP onshore (Figure 8)	Onboard	Onboard	Onshore	Yes	Delayed network outage	Medium
Core aboard (Figure 9)	Onboard	Onboard	Onboard	Yes	Local services survive	Highest

## Appendix A. Tables with Measurements Results

**Table A1:** Numerical values of the coverage plot at 3.5 GHz in Figure 2.

Distance	Received Signal Strength
(km)	(dBm)
0.0	−88.36
0.2	−95.66
0.5	−89.06
0.7	−94.72
1.0	−93.82
1.2	−106.36
1.5	−95.87
1.7	−97.75
2.0	−103.45
2.2	−100.4
2.5	−99.56
2.7	−109.6
2.9	−106.08
3.2	−102.97
3.4	−109.54
3.7	−98.43
3.9	−109.26
4.2	−105.5
4.4	−105.74
4.7	−108.77
4.9	−109.44
5.1	−107.94
5.4	−109.36
5.6	−109.4
5.9	−109.88
6.1	−106.78
6.4	−109.99
6.6	−108.74
6.9	−108.13
7.1	−109.83
7.4	−108.01
7.6	−105.92
7.8	−108.36
8.1	−109.72
8.1–8.4	Outage
8.4	−109.34
8.6	−108.35
8.6–9.4	Outage
9.4	−109.2
9.7	−109.04
9.9	−106.26
10.2	−108.38
10.4	−109.8
10.4–10.7	Outage
10.7	−109.41
10.7–11.5	Outage
11.5	−109.91
11.5–12.3	Outage
12.3	−109.13
12.3–17.1	Outage
17.1	−109.24

Coverage outages are considered for received signal power below −110 dBm.

**Table A2:** Numerical values of the coverage plot at 700 MHz in Figure 3.

Distance	Received Signal Strength
(km)	(dBm)
0.0	−105.2
1.0	−101.36
2.0	−104.63
2.9	−105.66
3.9	−103.1
4.9	−100.02
5.9	−102.39
6.8	−102.51
7.8	−100.17
8.8	−102.81
9.8	−101.52
10.7	−104.42
11.7	−96.96
12.7	−105.5
13.7	−100.2
14.6	−103.95
15.6	−103.75
16.6	−107.12
17.6	−107.03
18.5	−109.29
19.5	−109.29
20.5	−108.14
21.5	−107.45
22.4	−108.11
23.4	−106.25
24.4–29.3	Outage
29.3	−104.65
30.2	−107.8
31.2	−102.89
32.2	−105.73
33.2	−107.93
34.1	−108.85
35.1	−108.06
36.1	−109.37
37.1	−108.67
38.0	−107.87
39.0	−109.22
40.0	−102.84
41.0	−107.23
41.9	−104.32
42.9	−107.32
43.9	−109.1
44.9	−105.9
45.8	−108.73
45.8–47.7	Outage
47.7	−109.85
47.7–51.5	Outage
51.5	−109.53
51.5–55.5	Outage
55.5	−109.54
56.5	−106.8
57.5	−109.93
57.5–66.0	Outage
66.0	−109.27
66.0–73.3	Outage
73.3	−109.86
74.3	−109.3
74.3–77.2	Outage
77.2	−109.81
77.2–79.2	Outage
79.2	−109.51

Coverage outages are considered for received signal power below −110 dBm.
